# Oxidative Stress, Atherogenic Dyslipidemia, and Cardiovascular Risk

**DOI:** 10.3390/biomedicines11112897

**Published:** 2023-10-26

**Authors:** Jelena Vekic, Kristine Stromsnes, Stefania Mazzalai, Aleksandra Zeljkovic, Manfredi Rizzo, Juan Gambini

**Affiliations:** 1 Department of Medical Biochemistry, University of Belgrade-Faculty of Pharmacy, 11000 Belgrade, Serbia; jelena.vekic@pharmacy.bg.ac.rs (J.V.); aleksandra.zeljkovic@pharmacy.bg.ac.rs (A.Z.); 2 Department of Physiology, Faculty of Medicine, University of Valencia, 46010 Valencia, Spain; kristine.stromsnes@uv.es (K.S.); stefania.maz00@gmail.com (S.M.); juan.gambini@uv.es (J.G.); 3 Department of Health Promotion, Mother and Child Care, Internal Medicine and Medical Specialties, University of Palermo, 90100 Palermo, Italy

**Keywords:** free radicals, antioxidants, small dense LDL, remnant lipoproteins, non-cholesterol sterols, oxysterols

## Abstract

Oxidative stress is the consequence of an overproduction of reactive oxygen species (ROS) that exceeds the antioxidant defense mechanisms. Increased levels of ROS contribute to the development of cardiovascular disorders through oxidative damage to macromolecules, particularly by oxidation of plasma lipoproteins. One of the most prominent features of atherogenic dyslipidemia is plasma accumulation of small dense LDL (sdLDL) particles, characterized by an increased susceptibility to oxidation. Indeed, a considerable and diverse body of evidence from animal models and epidemiological studies was generated supporting oxidative modification of sdLDL particles as the earliest event in atherogenesis. Lipid peroxidation of LDL particles results in the formation of various bioactive species that contribute to the atherosclerotic process through different pathophysiological mechanisms, including foam cell formation, direct detrimental effects, and receptor-mediated activation of pro-inflammatory signaling pathways. In this paper, we will discuss recent data on the pathophysiological role of oxidative stress and atherogenic dyslipidemia and their interplay in the development of atherosclerosis. In addition, a special focus will be placed on the clinical applicability of novel, promising biomarkers of these processes.

## 1. Introduction

Reactive oxygen species (ROS) are present in all living systems; under physiological conditions, they can act as secondary signaling molecules. ROS have an important role in cell signaling, gene transcription, protein kinase activation, phosphatase inhibition, cell differentiation, apoptosis, cellular immunity, etc. [1,2]. However, under pathophysiological conditions, when the balance between the generation and the removal of free radicals is disrupted, increased levels of ROS contribute to cellular dysfunction through oxidative damage [3]. In aging, one of the causes of oxidative stress is a high rate of ROS generation through oxidative metabolism; it has been established that long-lived species show lower oxidative damage of their mitochondria due to a reduced production of ROS compared with other animals [4].

At the cellular level, the main source of ROS formation occurs in the mitochondria through cellular respiration. Specifically, the mitochondrial electron transport chain is the major source of ROS production. Other endogenous sources include nicotinamide adenine dinucleotide phosphate (NADPH) oxidase, myeloperoxidase, lipoxygenase, and xanthine oxidase [5]. In addition, many day-to-day biological processes, such as food digestion, metabolism of lipids and alcohol, as well as pollution, tobacco use, exposure to heavy or transition metals, and radiation produce free radicals [6]. To combat oxidation, our cells developed detoxifying mechanisms called antioxidants. These can be of the enzymatic or non-enzymatic type. In addition, antioxidants can be incorporated from exogenous sources, either through food or dietary supplements (Table 1).

Enhanced production of ROS, whether endogenous or exogenous, and/or decreased efficiency of the antioxidant defense system cause oxidative stress, further leading to oxidative modification of the major cellular macromolecules. It is now firmly established that the detrimental consequences of oxidative stress play an important role in the development of numerous diseases. Accordingly, lipid peroxidation is considered the earliest event in atherogenesis. The major targets of lipid peroxidation are unsaturated fats and cholesterol, including low-density lipoprotein (LDL) particles, particularly small, dense LDL (sdLDL), which are highly vulnerable to oxidation. The accumulation of sdLDL particles in the plasma is a common feature of atherogenic dyslipidemia, which is a specific alteration of lipoprotein metabolism driven by insulin resistance. It is characterized by both quantitative and qualitative changes in plasma lipoproteins. Insulin resistance is accompanied by hyperglycemia, as well as by a progression of pro-oxidant and pro-inflammatory changes [7], and these processes altogether increase the possibility of adverse modifications of plasma lipoproteins. The above-mentioned alterations exert an important role in promoting atherogenesis, not only through the formation of foam cells but also through their direct effects on vascular cells. LDL oxidation is a complex process during which both its protein and lipid content undergo oxidative changes that facilitate cholesterol accumulation in subendothelial macrophages [5]. Other important targets are poly-unsaturated fatty acids (PUFAs), particularly linoleic and arachidonic acids [8]. 

The oxidative stress theory of aging is based on the hypothesis that age-associated functional losses are due to the accumulation of oxidative damage to macromolecules. Although the exact mechanism of aging induced by oxidative stress is still unclear, one of the causes is believed to be increased ROS production leading to cellular senescence. These senescent cells acquire an irreversible senescence-associated secretory phenotype that involves the secretion of soluble factors, such as interleukins, chemokines, growth factors, degrading enzymes, and insoluble proteins/extracellular matrix components [9]. With the close relationship between oxidative stress, inflammation, and aging in mind, the oxidation–inflammatory or oxy-inflammation–aging theory of aging has been proposed. The activation of the immune system due to disrupted redox homeostasis induces an inflammatory state that creates a vicious cycle in which chronic oxidative stress and inflammation feed off of each other and consequently increase age-related morbidity and mortality [10]. Cardiovascular disease (CVD) is one of the main causes of morbidity and mortality in the elderly, and atherosclerosis plays a crucial role as the main causative event. Several studies have shown that the tolerance of the heart to oxidative stress decreases with age due to a reduction in the concentrations of antioxidant enzymes, which contributes to the development of cardiovascular disorders, driven by an increase in the oxidation of plasmatic lipids, among other causes [11]. This paper will discuss the interplay between oxidative stress and atherogenic dyslipidemia in atherogenesis, based on the available data gathered from recent studies and emphasizing the role and clinical applicability of newly proposed biomarkers. 

## 2. The Role of Oxidative Stress in the Development of Atherosclerosis

**Figure 1:**
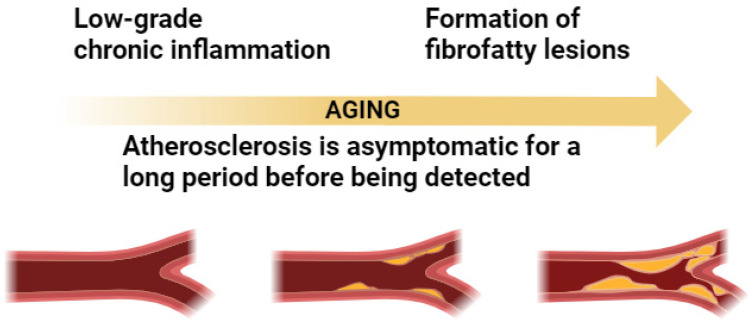
Relation between atherosclerosis and inflammation in aging.

Atherosclerosis is a disease caused by a low-grade, chronic inflammation of the arterial wall. It is characterized by the formation of fibrofatty lesions that begin early in life and progress gradually and is therefore usually asymptomatic for a long period before being detected (Figure 1). 

**Figure 2:**
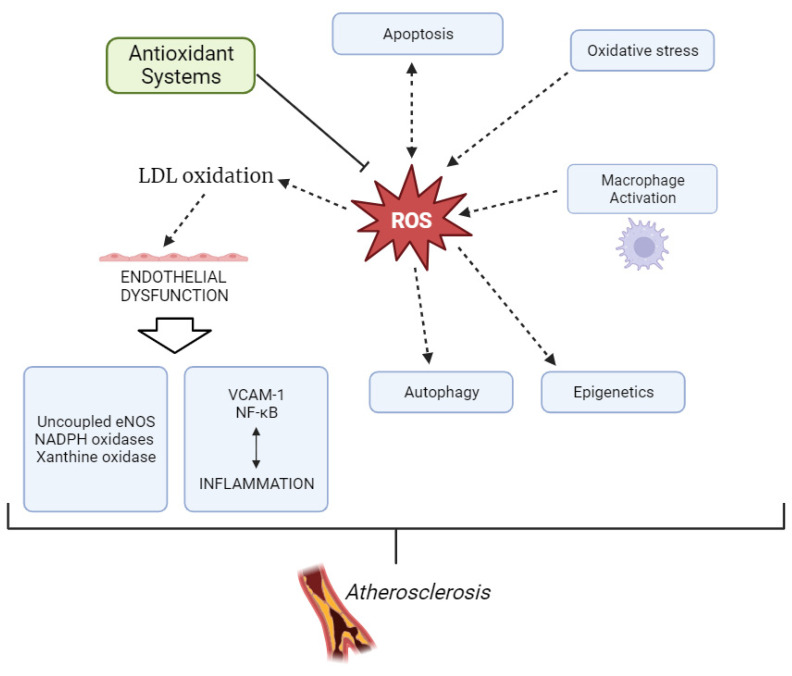
Development of atherosclerosis through overproduction of ROS.

The formation of atherosclerotic plaque is initiated by the accumulation of LDL particles, especially sdLDL, and fibrous elements in medium and large arteries, such as the aorta, carotid, and coronary arteries [12,13]. However, to become atherogenic, LDL needs to undergo chemical modifications, with the most studied being desialylation and oxidation. As recently reviewed by Bale et al. [14], virtually all traditional CVD risk factors are able to trigger ROS production or mediate the process of lipoprotein oxidation. Oxidized LDL (ox-LDL) accumulates in predisposed areas of the arterial wall and contributes to an increased expression of cell adhesion molecules on the endothelial cells, such as vascular cell adhesion molecule-1 (VCAM-1). Activation of endothelial VCAM-1 expression is driven by inflammatory signaling, which causes antioxidant-inhibiting mechanisms, involving a redox-sensitive activation of nuclear factor kappa-light-chain-enhancer of activated B cell (NF-κB) [11]. In addition to ox-LDL and inflammatory mediators, recent data have suggested other mechanisms that are critically important for oxidative stress exacerbation in atherosclerosis, including macrophage cellular oxidation and apoptosis, as well as the role of autophagy and epigenetic processes [15,16]. Therefore, oxidative stress has been shown to play a major role in the pathogenesis of atherosclerosis (Figure 2) [17]. In addition to atherosclerotic cardiovascular disease, more recent data suggest that oxidative stress is implicated in the pathogenesis of left ventricular hypertrophy, diastolic dysfunction, heart failure, and ischemia/reperfusion injury [18].

ROS are involved in atherosclerosis through several mechanisms. Apart from the production of oxidized lipoproteins, ROS also cause direct damage to the cellular and nuclear membranes and interact with endogenous vasoactive mediators in endothelial cells [19,20]. The vascular wall contains both antioxidant and oxidant systems. The oxidant system includes lipoxygenases, mitochondrial respiratory chain enzymes, xanthine oxidase, uncoupled eNOS, and NADPH oxidases (Nox), among others. The antioxidant system is comprised of superoxide dismutase (SOD), glutathione peroxidases, catalase, peroxiredoxins, paraoxonases (PONs), the thioredoxin system, etc. Nox is considered one of the main sources of ROS at the vascular wall. Its increased activity leads to eNOS uncoupling, which reduces NO bioavailability and thereby leads to endothelial dysfunction. Additionally, uncoupled eNOS produces O_2_^−^, thereby further aggravating vascular oxidative stress. The main causes of eNOS uncoupling are related to ox-LDL, eNOS S-glutathionylation, L-arginine or tetrahydrobiopterin deficiencies, and hyperglycemia [13]. 

The antioxidant system comprises enzymes like superoxide dismutase (SOD), glutathione peroxidases, catalase, peroxiredoxins, paraoxonases (PONs), the thioredoxin system, etc. Most of the antioxidant enzymes, including glutathione peroxidases, reduce non-radical oxidants, especially organic hydroperoxides. Peroxiredoxins catalyze the reduction of organic hydroperoxides, H_2_O_2_, and peroxynitrite. Catalase decomposes hydrogen peroxide as well as glutathione peroxidase, which further reduces lipoperoxides. However, superoxide dismutase provides a cellular defense against ROS, catalyzing the dismutation of O_2_^−^ superoxide radicals to O_2_ molecular oxygen.

Macrophages are known as scavenger cells for available ox-LDL. The oxidative modification of LDL leads to macrophage uptake and cellular accumulation of cholesterol. The macrophages phagocytosing ox-LDL are called foam cells, due to their lipid-like appearance [21,22,23]. The presence of these foam cells in the arterial wall is a hallmark of early atherosclerotic lesions [24,25]. In addition to macrophage migration, mast cells and T-lymphocytes are also recruited to the arterial wall in response to oxidative stress. The increased presence of these immune cells leads to the release of cytokines, thereby inducing inflammatory processes and ROS production. For instance, IL-1β has been shown to induce the production of ROS by NADPH oxidase; TNF-α induces the production of mitochondrial ROS; and IFN-γ can induce ROS production through both the mitochondrial and NADPH oxidase pathways [26,27]. Additionally, Bruton’s tyrosine kinase (BTK) has been found to regulate the inflammatory responses of macrophages in atherosclerosis. In a recent study, knockdown of BTK was found to impede ox-LDL-induced NF-*κ*B signaling activation in macrophages, as well as mitochondrial injury, and oxidative and endoplasmic reticulum stress [28].

Furthermore, the activation of the MAPK signaling pathway is closely related to atherosclerosis. This pathway comprises c-Jun N-terminal protein kinase (JNK), extracellular signal-regulated kinase (ERK), and p38 mitogen-activated protein kinase (p38MAPK) [29]. This pathway is affected by oxidative stress and the expression of pro-inflammatory factors in endothelial cells, which induces the development of atherosclerosis. The MAPK signaling pathway is activated mainly by ox-LDL, causing MAPK phosphorylation in the blood which produces abundant ROS, thereby promoting monocyte accumulation in the arterial wall, and a reduction in the secretion of collagen and other components of the extracellular matrix by the vascular smooth muscle cells, which ultimately elicits cytotoxicity [30]. This leads to foam cell necrosis in the vascular plaque, resulting in atherosclerotic plaque fragmentation and eventually the formation of thrombi in blood vessels [31]. Additionally, in a recent study by Tabas et al. [32], pro-inflammatory macrophage activation in *Jak2VF* mice was found to exacerbate atherosclerosis due to impaired efferocytosis of apoptotic cells by phagocytes, via p38 MAP kinase and by pro-inflammatory cytokine and chemokine production.

Mechanisms of cross-regulation have been reported between these pro-oxidant systems. According to the theory of “kindling-bonfire radicals”, ROS sources can be classified into two groups: the initial group, consisting of the mitochondrial respiratory chain and NADPH oxidases, and a secondary group including xanthine oxidase and uncoupled eNOS [11]. The crosstalk between these two groups of enzymes occurs through the generation of ROS. ROS produced by the initial source trigger the activation of secondary sources. Therefore, this “kindling-bonfire” theory postulates that the primary, NADH oxidase-derived, ROS kindle the production of ROS by secondary sources, which subsequently kindles a tertiary source, believed to be the mitochondria. This results in a “bonfire” of radicals and oxidative stress [11,33]. The resulting cascade of ROS production has been identified as an important mechanism underlying human inflammatory disorders as an inflammasome activator [34]. 

In recent years, special attention has been paid to the transcription factor Nrf2 and its downstream-regulated protein heme oxygenase-1 as protectors against atherosclerotic injury [35]. Nrf2 belongs to the “Cap’n’Collar” family of transcription factors that modulate the cellular redox status [36]. Under non-stressful conditions, Nrf2 is trapped in the cytosol by Kelch-like ECH-associated protein-1 (KEAP-1) [37]. However, ROS, ox-LDL, lipid peroxides, pro-inflammatory cytokines, and other molecules related to oxidative stress induce an alteration of KEAP-1 conformation, thereby promoting the release of Nrf2, which then translocates to the nucleus. Once in the nucleus, it can modulate the expression of genes encoding antioxidant proteins, as well as regulate the thioredoxin and glutathione systems, iron homeostasis, and NADPH production and utilization [38,39]. The thioredoxin system has also been proposed as a therapeutic target for atherosclerosis [12]. Thioredoxin-1 is a small protein normally found in mammalian cells that responds to changes in the redox environment by regulating redox-related proteins or contributing electrons [40]. In a recent study, thioredoxin-1 was found to inhibit the ROS-activated NLRP3 inflammasome, which plays an important role in inflammation regulation, through the secretion of caspase-1, p10, and IL-1β in vitro [14]. Additionally, in a mouse model of atherosclerosis, thioredoxin-1 inhibited atherosclerosis development [41]. Furthermore, recombinant human thioredoxin-1 has been found to suppress ox-LDL-stimulated macrophage apoptosis and foam cell formation through the inhibition of ROS generation, LOX-1 expression, and p38 MAPK activation [42]. 

## 3. The Role of Atherogenic Dyslipidemia in the Development of Atherosclerosis

Atherogenic dyslipidemia is a common finding in patients with cardiometabolic diseases, such as metabolic syndrome and type 2 diabetes mellitus (DM) [43]. In addition to lipid disorders, these conditions are usually characterized by multiple metabolic abnormalities, such as obesity, hyperglycemia, and low-grade inflammation [7,44]. Based on the main alterations in lipid profile, comprising increased TG levels, reduced concentrations of high-density lipoprotein cholesterol (HDL-C), and elevated sdLDL particles, this complex form of dyslipidemia is also recognized as the lipid triad or atherogenic lipoprotein phenotype [45,46]. Alternatively, this lipid pattern is referred to as “metabolic dyslipidemia”, according to the major underlying pathophysiological mechanism [47]. Finally, due to a high prevalence in patients with DM, it is also frequently termed diabetic dyslipidemia [48]. 

The main pathophysiological mechanisms that underlie the alteration in lipoprotein metabolism within atherogenic dyslipidemia are driven by the lack of insulin activity due to insulin resistance and/or relative insulin deficiency. Such a metabolic environment stimulates the process of lipolysis in adipose tissue and the consequent excessive delivery of free fatty acids to the liver for de novo lipogenesis [49], resulting in enhanced formation and secretion of very-low-density lipoproteins (VLDLs) [50]. Of note, an increased influx of fatty acids into the liver is also a trigger for hepatic steatosis development [49]. Reduced clearance of chylomicrons and VLDL particles, due to inhibition of the synthesis and activity of lipoprotein lipase (LPL), is another putative mechanism for the development of atherogenic dyslipidemia [51]. In hypertriglyceridemia, the activity of cholesteryl ester transfer protein (CETP) facilitates the exchange of core lipids between TG-rich lipoproteins (TRLs), and LDL and HDL particles [52]. Finally, the activity of hepatic lipase (HL) generates sdLDL, as well as small HDL particles, which are more rapidly catabolized, leading to decreased HDL-C levels [46]. 

There is much evidence demonstrating that patients with atherogenic dyslipidemia are at an increased CVD risk. This association is supported not only by the results of observational studies [53], but also in clinical trials [43,54]. Atherogenic dyslipidemia is a complex, multifactorial trait, but due to the limited availability and the cost of sdLDL determination, its contribution to CVD risk is usually estimated through a concomitant presence of hypertriglyceridemia and low HDL-C levels. In line with the previous statement, recent data from the Look AHEAD study showed that atherogenic dyslipidemia in patients with DM was associated with a significantly higher risk of cardiovascular events as compared to patients without lipid disorders after a 9.5-year follow-up analysis [55]. Furthermore, prospective data from the Renal Insufficiency and Cardiovascular Events (RIACE) cohort showed an independent association of atherogenic dyslipidemia with all-cause mortality in type 2 DM patients [56]. This characteristic lipid pattern is also associated with residual vascular risk in stroke survivors [57] and was recently described in severely ill COVID-19 patients [58]. 

One of the hallmarks of atherogenic dyslipidemia is an increase in TRLs, in both fasting and postprandial states [59,60]. In the last decade, significant progress has been made to better understand the pathophysiological roles of TRLs [61]. This renewed interest in TG metabolism provided firm evidence that TRLs contribute to the increased CVD risk associated with atherogenic dyslipidemia and revealed potential new therapeutic targets [62]. An overproduction and/or impaired lipolysis and clearance of TRLs results in intensive intravascular remodeling and the accumulation of cholesterol-enriched remnant particles in the plasma. The mechanisms conferring increased atherogenicity of remnant lipoproteins include direct deposition of cholesterol into the vascular wall and pro-inflammatory and pro-oxidative properties [63,64]. Thus, remnant cholesterol content is considered the main determinant of the risk associated with TRLs. The results of the Women’s Health Study showed that elevated remnant cholesterol at baseline was associated with incident CVD during the 15.7 years of follow-up [65]. Furthermore, remnant cholesterol was related to a residual cardiovascular risk among statin-treated patients [66]. A recent study by Gao et al. [67] showed that elevated remnant cholesterol was associated with a risk of major adverse cardiovascular events in patients with myocardial infarction with nonobstructive coronary arteries. Similarly, prospective data from the Copenhagen General Population Study, including 87,192 subjects with 13 years of follow-up, showed that remnant cholesterol higher than 1 mmol/L was associated with two-fold increased mortality from cardiovascular disease and other causes [68]. 

**Figure 3:**
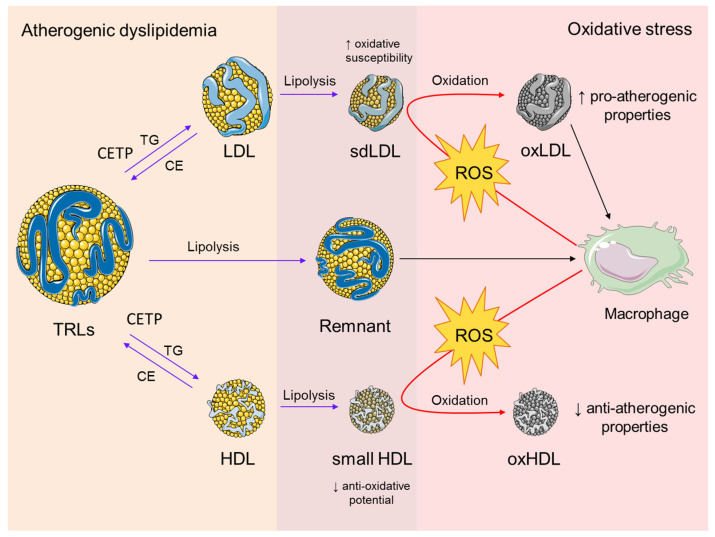
The interplay between atherogenic dyslipidemia and oxidative stress. Atherogenic dyslipidemia is characterized by increased plasma levels of TRLs. It is also associated with enhanced activity of CETP, which mediates the exchange of core TGs in TRLs for CE in LDL and HDL particles. A subsequent process of lipolysis results in the formation of sdLDL and small HDL particles with increased oxidative susceptibility and reduced anti-oxidative potential, respectively. The lipolysis of TRLs generates remnant particles, which are capable of accumulating in the macrophages in their native form. Enhanced production of ROS by the macrophages induces oxidative modifications of sdLDL and HDL particles. The main pro-atherogenic effect of oxLDL particles is reflected by their preferential uptake by macrophages and consecutive foam cell formation, while oxHDL has compromised anti-atherogenic properties. Both oxidized LDL and HDL particles act synergistically in the maintenance of a pro-oxidative state during the progression of atherosclerosis. Abbreviations: TRLs, triglyceride-rich lipoproteins; CETP, cholesteryl ester transfer protein; TG, triglyceride; CE, cholesteryl esters; sdLDL, small dense LDL; ROS, reactive oxygen species, oxLDL, oxidized LDL; oxHDL, oxidized HDL. The figure was composed using Servier Medical Art templates, licensed under a Creative Common Attribution 3.0 (https://smart.servier.com, accessed on 22 August 2023).

Novel data showed that cardiometabolic disorders are accompanied by changes in the lipidome and proteome of TRL particles [69,70]. In patients with type 2 DM, the process of non-enzymatic glycation of apolipoproteins within TRLs is also pronounced [71], and each of the above-mentioned structural modifications might affect the metabolism of TRLs and increase their pro-atherogenic potential. A significant contribution to the link between TRLs and oxidative stress has been provided by studies investigating the postprandial response to a high-fat meal. And, it is believed that an increase in postprandial TRLs may cause endothelial dysfunction through the induction of oxidative stress [72,73]. It has also been demonstrated that VLDL particles isolated from patients with metabolic syndrome can induce ROS-mediated apoptosis of endothelial cells [74]. Finally, it was confirmed that the oxidation of free fatty acids, released during the lipolysis of TRLs, exerts pro-inflammatory effects [75]. Taken together, these data indicate that TRLs and oxidative stress have interactive roles in atherosclerosis (Figure 3).

As mentioned earlier, elevated TRLs promote adverse remodeling of LDL particles and enhanced formation of smaller and denser LDL species (Figure 3). The role of sdLDL in the development of atherosclerosis was recently reviewed by several independent groups [46,76,77,78,79,80], thus providing new evidence for their clinical importance as a risk factor and therapeutic target. Nowadays, a consensus has been reached among lipidologists that information on sdLDL levels would enable a residual risk assessment and better management of patients [78,79]. One of the most prominent features that contribute to an increased atherogenicity of sdLDL particles is their susceptibility to in vitro oxidation [81]. An enhanced susceptibility of sdLDL to oxidation is mainly attributable to its altered surface lipid composition, reflected by a reduced content of free cholesterol and increased content of PUFAs. Also, sdLDL particles bear larger amounts of lipid peroxidation products, but a lower content of antioxidants [46]. In addition, sdLDL particles are also prone to non-enzymatic glycation, while glycated LDLs are more sensitive to oxidative modification [82]. Earlier studies demonstrated that insulin-resistant subjects have elevated plasma ox-LDL particles [83,84,85]. Yet, it was only recently confirmed that high sdLDL levels in the plasma were also associated with increased levels of ox-LDL particles in patients with CVD [86], as well as in the general population [87]. As mentioned earlier, lipid peroxidation of LDL particles results in the formation of various bioactive lipid species [88] that contribute to the atherosclerotic process through different pathophysiological mechanisms, including foam cell formation, direct detrimental effects, and receptor-mediated activation of pro-inflammatory signaling pathways. In accordance with this, a soluble lectin-like oxidized low-density lipoprotein receptor-1 (LOX-1) emerged as a novel diagnostic and prognostic biomarker of acute coronary syndrome [89,90].

Current research has revealed numerous overlaps between oxidative stress and dyslipidemia during the development of atherosclerosis, one of them being related to oxysterols. Recently, much attention has been given to the possible role of oxysterols in the onset and progression of atherosclerotic plaques. Oxysterols are products of enzymatic and non-enzymatic oxidative modifications of cholesterol and other sterols [91,92]. Non-enzymatic oxidation is generally driven by ROS. Oxidation of the steroid moiety occurs within the process of LDL oxidation in the sub-endothelium, wherein 7-ketocholesterol is the major product, but cholesterol and other sterols can undergo oxidation in cells and plasma as well. Importantly, a significant source of oxysterols in the human body is through the intestinal absorption of processed food of animal origin [91]. It has been shown that plasma oxysterol levels are increased in obese subjects and individuals with metabolic syndrome when compared to healthy individuals, and the observed differences were gender-related [93]. In addition, plasma oxysterols positively correlated with early signs of microvascular dysfunction in apparently healthy examinees [94]. 7-ketocholesterol, the most abundant oxysterol in atherosclerotic plaques, is reported to be positively associated with unfavorable cardiovascular outcomes in patients with coronary artery disease [95]. Similarly, higher levels of specific oxysterols were found in the plasma and arterial tissue of patients with peripheral atherosclerotic disease, in comparison to healthy individuals [96]. Interestingly, the results of Dias et al. revealed that simvastatin treatment significantly decreased plasma levels of free oxysterols generated by non-enzymatic ROS-induced oxidation, thus suggesting another possible beneficial effect of statins in subjects with hypercholesterolemia [97].

The mechanisms by which oxysterols contribute to atherosclerosis development are not fully understood. Recently, it was hypothesized that the elevated presence of oxysterols in the plasma membrane of endothelial cells can provoke defects in membrane phospholipid assembly and thus enhance the transport of LDL into the subendothelial space and initiation of atherosclerotic lesions [98]. Moreover, it has been demonstrated that 7-ketocholesterol induces mitochondrial dysfunction and subsequent apoptosis of macrophages, which is an important step in the formation of unstable atherosclerotic plaques [99]. Additionally, 7-ketocholesterol reportedly promotes leukocyte–endothelial cell interactions by upregulating the expression of E-selectin, ICAM-1, and VCAM-1 proteins [100]. Likewise, it has been shown that this oxysterol stimulates the formation and activation of the NLRP3 inflammasome in cultured carotid endothelial cells, which contributes to vascular injury [101]. Interestingly, although a pro-atherosclerotic role was confirmed for endogenous oxysterols, such as 7-ketocholesterol, similar conclusions cannot be drawn for oxyphytosterols. Namely, an in vitro study did not find evidence that oxyphytosterols influence macrophage-mediated inflammation [102]. Furthermore, analysis of data from the Framingham Offspring Study did not reveal any associations between plasma levels of oxyphytosterols and increased CVD risk [103]. Thus, the role of oxidized phytosterols remains to be elucidated.

## 4. Biomarkers of Oxidative Stress and Atherogenic Dyslipidemia: Current Perspective and Future Directions

To date, numerous products of the oxidation of nucleic acids, proteins, carbohydrates, and particularly lipids have been suggested as biomarkers for increased cardiovascular risk. In addition, the enzymes responsible for the generation or degradation of free radicals can also be considered redox biomarkers. It has been shown that these biomarkers provide information on CVD risk and disease severity but could also serve as predictors of mortality. In general, lipid peroxidation can generate different types of oxidized species such as isoprostanes, epoxides, oxysterols, chlorohydrins, and nitro compounds, among others. Hydroperoxides are produced mainly by the oxidation of PUFAs, commonly present within cholesteryl- and phospholipid esters, and can be detected in urine. Furthermore, the analysis of lipid hydroperoxides isomers provides information on the mechanisms involved in the process of lipid peroxidation, which might facilitate selection of an appropriate antioxidant therapy. Recently, Kato et al. [104] introduced a novel method for 1-palmitoyl-2-linoleoyl-*sn*-glycero-3-phosphocholine hydroperoxide (PC 16:0/18:2;OOH) and cholesteryl linoleate hydroperoxide (CE 18:2;OOH) isomer determination by liquid chromatography–tandem mass spectrometry (LC-MS/MS). Based on the isomeric profile, the authors concluded that radical and enzymatic oxidation were the predominant routes of phospholipid and cholesteryl ester peroxidation within LDL and HDL particles from healthy subjects [104]. In addition to ox-LDL particles, plasma malondialdehyde (MDA) and 4-hydroxynonenal (HNE), as well as urinary 8-iso-prostaglandin F2α (8-iso-PGF2α), are currently the most widely studied biomarkers in terms of CVD prediction and prognosis. At this point, it should be mentioned that hydroxyl and organic peroxyl radicals are able to induce protein peroxidation, resulting in protein hydroperoxide formation. Yet, their characterization and quantification are more difficult than in the case of lipid hydroperoxides.

Despite a long-term interest in raising HDL-C levels, current clinical practice guidelines consider low HDL-C levels as a CVD biomarker, but not a target for therapy [105]. This is in part due to the highly heterogeneous nature of HDL particles, in terms of their shape, size, and composition, that arises because of continuous intravascular remodeling in both physiological and pathophysiological states. In atherogenic dyslipidemia, the lipid content of HDL particles is significantly altered and characterized by a decrease in cholesteryl esters and a concomitant increase in TG content [106]. Furthermore, oxidative stress might enhance lipid peroxidation within HDLs, leading to the accumulation of oxidized HDL particles in the plasma of patients with cardiometabolic diseases [107,108]. Of note, these patients usually have reduced activity of paraoxonase-1 (PON1), the main antioxidative enzyme of HDLs [109,110]. Finally, the accompanying pro-inflammatory state may alter the protein composition of HDL particles, by increasing their serum amyloid A (SAA) content [111]. The above-mentioned alterations in HDL structure affect its capacity for reverse cholesterol transport and other protective properties [112]. Taking all this into account, it is obvious that plasma HDL-C level does not reflect HDL’s structure and dysfunctionality in cardiometabolic and other diseases. Therefore, one of the tasks of future studies should be to evaluate the usefulness of novel indices for HDL characterization, such as cholesterol efflux capacity [113] or HDL inflammatory index [114], which are less explored in patients with cardiometabolic diseases.

Apart from limited efficacy in primary prevention, the use of LDL-C levels alone in predicting the risk of recurrent CVD events might be inefficient, especially in patients treated with innovative lipid-lowering therapy which lower LDL-C levels [115]. These observations indicate that the ability to early recognize increased CVD risk and thus enable timely prevention in several categories of high-risk patients depends on advanced lipid testing. Therefore, finding reliable biomarkers of LDL heterogeneity in the future would have great benefit for the early diagnosis and management of patients. Over the past decades, several advanced laboratory methods, such as ultracentrifugation, gradient gel electrophoresis, or nuclear magnetic resonance, were employed for sdLDL assessment. However, their implementation in routine medical laboratories and subsequent application in clinical practice requires further standardization and normalization [116]. So far, several biomarkers have been used for sdLDL characterization, including sdLDL-cholesterol (sdLDL-C) level, and LDL particle number (LDL-P) and size, and they are generally comparable in terms of CVD prediction [117]. In parallel, certain lipid ratios, particularly TG/HDL-C [118], TG/LDL-C [119], or apoB/LDL-C [120], have been suggested as suitable alternative indices of increased sdLDL particles in the plasma. From the current perspective, sdLDL-C seems to have the greatest potential for clinical application in both primary and secondary prevention [117,121].

**Figure 4:**
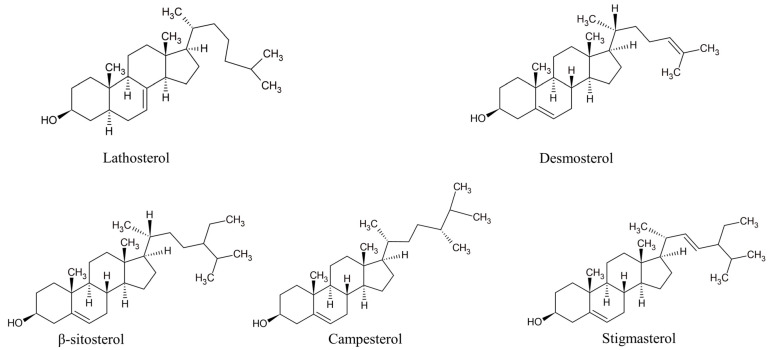
Structures of prominent non-cholesterol sterols. Lathosterol and desmosterol—cholesterol synthesis markers; β-sitosterol, campesterol, and stigmasterol—cholesterol absorption markers.

Nowadays, it is widely accepted that a full understanding of changes in the lipid status during the development of atherosclerosis requires an insight into the metabolism of the major lipid component—cholesterol. Cholesterol homeostasis is largely based on the balance between two processes: its endogenous synthesis from non-steroid precursors and the intestinal absorption of dietary cholesterol. Circulatory endogenous precursors of cholesterol can be used as markers of cholesterol synthesis, while phytosterols, which use the same absorption route as cholesterol, are proposed as cholesterol absorption markers (Figure 4) [122]. Cholesterol synthesis and absorption are reciprocally regulated in healthy individuals, but it has been demonstrated that various pathological states are characterized by the disturbance of the balance between these processes [123,124,125,126,127,128,129,130]. Accordingly, non-cholesterol sterols could be used, not only as indicators of altered cholesterol homeostasis, but also as markers of several diseases, including CVD, and predictors of therapeutic response. However, it should be noted that these hypotheses are not univocally confirmed [131,132,133]. Non-cholesterol sterols have also been analyzed concerning their plausible roles as signaling molecules. It has been shown that the cholesterol precursor desmosterol interferes in the formation of atherosclerotic lesions, by affecting both cholesterol synthesis and inflammatory responses in macrophages [134]. Similarly, desmosterol, by interacting with liver X receptors, exhibits anti-inflammatory effects and enables the repair of demyelinated lesions in multiple sclerosis [135]. Apart from the well-known role of phytosterols in the reduction of intestinal cholesterol absorption, the available evidence suggests that β-sitosterol ameliorates insulin resistance and inflammation by targeting insulin receptor signaling [136,137] and IKKβ/NF-κB and JNK signaling in adipose tissues [138]. Specific antioxidant, anti-inflammatory, anti-diabetic, and anti-cancer effects have been demonstrated for stigmasterol [139] as well as for campesterol [140,141]. Thus, non-cholesterol sterols should be regarded not merely as biomarkers of overall cholesterol metabolism, but as unique lipid species capable of independently activating specific mechanisms that are involved in the process of atherogenesis.

In recent years, additional molecular mechanisms have been suggested to affect the development of oxidative stress and dyslipidemia in patients with cardiometabolic disorders, such as microRNAs [47,142], telomere length [143], and DNA methylation [46]. In addition, the era of “omics” technologies provides almost unlimited possibilities to identify novel biomarkers and therapeutic targets [144], which will hopefully result in a more individualized approach for risk assessment and therapy. Among them, lipidomics represents a powerful research tool for the identification of candidate lipid biomarkers, such as different phospholipid species [145,146], but their clinical importance remains to be verified in the future. 

## 5. Conclusions

Oxidative stress and atherogenic dyslipidemia play an interactive role in the development of atherosclerosis. Thus, one of the promising approaches in the management of atherosclerotic risk is to simultaneously target multiple dysregulated metabolic pathways, including the formation and clearance of both ROS and proatherogenic lipoprotein particles. In this respect, the prevention of vascular oxidative stress may be an effective therapeutic strategy against cardiovascular risk factors, atherosclerosis, and atherogenic dyslipidemia. Since the results of clinical trials with antioxidants are largely inconclusive [15], positive changes in lifestyle habits and regular control of traditional CVD risk factors seem to be the most efficient preventive measures to halt both oxidative stress and atherosclerosis [14]. Modern management of atherogenic dyslipidemia involves both pharmaceutical and nutraceutical-based approaches [147]. Yet, the need for the optimization of the therapy by a more personalized approach was recently recommended by the International Lipid Expert Panel [148]. Although such treatments have already shown promising results, further clinical trials are warranted.

## Figures and Tables

**Table 1:** Endogenous and exogenous antioxidants.

	Antioxidants	
Endogenous Enzymatic	Endogenous Non-Enzymatic	Exogenous Non-Enzymatic
Superoxide dismutase copper	Glutathione	Carotenoids
Thioredoxin reductase	Uric acid	Lipoic acid
Glutathione peroxidase	Metal-binding proteins	Vitamin C
Catalase	Bilirubin	Vitamin E
Selenoprotein	Polyamines	Polyphenols
Peroxiredoxin	Coenzyme Q10	Hydroxycinnamic acids

## Data Availability

No new data were created or analyzed in this study. Data sharing is not applicable to this article.
